# Understanding the Broader Impacts Of Alcohol Marketing: Time For a Research Agenda Which Includes Adults

**DOI:** 10.1093/alcalc/agab028

**Published:** 2021-04-09

**Authors:** Nathan Critchlow, Crawford Moodie

**Affiliations:** Institute for Social Marketing and Health, Faculty of Health Sciences and Sport, University of Stirling, Stirling, FK9 4LA, Scotland

## Abstract

Most research on alcohol marketing involves young people. Consequently, gaps remain in our understanding of how alcohol marketing reaches, engages and influences adults, who are the legitimate and primary targets for marketing communications. Responding to these lacunae in knowledge is necessary to help inform and evaluate population-level controls on alcohol marketing.

The extant research literature on alcohol marketing predominantly involves studies with children and adolescents, with this evidence having shaped our understanding of the impacts of alcohol marketing (e.g. Anderson *et al.*, 2009; Jernigan *et al.*, 2017) and policies aimed at safeguarding youth (e.g. restrictions on alcohol advertising in youth-targeted media). While a focus on vulnerable populations, such as young people, is clearly appropriate, it should not be at the expense of adults. After all, in countries where alcohol marketing is permitted, adults who meet the minimum legal drinking age are the only legitimate targets for any promotional efforts, from mass media advertising to sponsorship and fully branded packaging, and they are the primary source of revenue for alcohol companies.

With alcohol consumption being commonplace in many parts of the world, the size of the adult market is extensive. For example, more than half of those aged ≥15 years in the European (59.9%), Americas (54.1%) and Western Pacific regions (53.8%) are current drinkers (World Health Organization, 2018). As such, examining how commercial activities shape and reinforce alcohol use and attitudes among adults is critical, particularly as it is estimated that, globally, two-fifths (39.5%) of drinkers have engaged in heavy episodic drinking (≥60 g of pure alcohol on at least one single occasion at least once per month) (World Health Organization, 2018), and adults experience, or contribute to, myriad individual, social and economic alcohol-related harms (World Health Organization, 2018).

Among a range of arguments against marketing regulation, alcohol companies contend that marketing activities are intended to retain existing adult customers and facilitate switching among adult drinkers not using their brands, and there is no evidence to support a causal effect on overall consumption or higher-risk drinking (Savell *et al.*, 2016; Martino *et al.*, 2017). Critical appraisals of alcohol marketing campaigns, however, suggest otherwise (Hastings *et al.*, 2010; Maani Hessari *et al.*, 2019). It is time these diametrically opposed standpoints were explicitly tested. While econometric studies offer some insight into the effect of marketing on adults (Saffer, 2020), the focus is typically on advertising rather than on the broader marketing mix, which includes, but is not limited to, other forms of promotion (e.g. product placement, celebrity endorsement, competitions, etc.) as well as price, place and product. In addition, the outcome measures in econometric research seldom reflect consumer-level indicators of harm, such as progression to heavy episodic or hazardous drinking and changes in attitudes or expectancies around drinking. That increased marketing expenditure is accompanied by sales and revenue growth—a relationship acknowledged in alcohol industry discourse (Glenday, 2019)—is testament to the role that marketing plays in stimulating consumption among the target adult market, but it offers no understanding as to what aspects of marketing are most effective and how they shape consumption.

Consequently, there remain fundamental research questions about the relationship between adults and alcohol marketing which require new insight or further elaboration. Where, and how often, do adults see marketing? Is marketing causally linked to increased consumption among adults and, if so, what types of consumption patterns and what levels of risk? How does marketing shape or reinforce norms, attitudes and cognitions around alcohol among adults? Does exposure to, and the effect of, marketing vary by key demographics? What is the relationship between alcohol marketing and health and social inequalities? Does marketing disproportionately affect some adults, for example, heavier drinkers or those in recovery (Guillou-Landreat *et al.*, 2020)? This is not intended to be an exhaustive list, but an indicator of the lacunae in knowledge.

Our recommendation for an increased emphasis on adults in research on alcohol marketing is echoed elsewhere. For example, Meier (2011) called for research to examine whole population effects of alcohol marketing, greater recognition of the complexity of how marketing activities influence existing drinkers (e.g. before, during and after consumption) and examination of the psychological processes that explain the effect of marketing. Atkinson *et al.* (2019) examined how women are targeted and represented in alcohol marketing, but highlighted a lack of research examining women’s perceptions of such marketing and the impact on their alcohol-related attitudes and consumption, including among adult consumers. Walls *et al.* (2020) highlight that there remains a paucity of research into the growing presence and influence of alcohol marketing in low- and middle-income countries; it may not be possible or wise to extrapolate the findings from studies in high-income countries to emerging markets.

The role of alcohol marketing on adult drinking behaviors is also policy relevant. The World Health Organization cites statutory controls on advertising as a best buy in reducing alcohol-related harm (World Health Organization, 2013). Some countries (e.g. France and Norway) have longstanding controls on where alcohol advertising can be placed and the types of messaging permitted (e.g. limited to only factual information about the product), while others have either recently implemented controls (e.g. Lithuania and the Republic of Ireland) or are assessing the need for new restrictions (Scottish Government, 2018). While these controls provide some targeted protection to younger consumers, they also have population-level impacts on marketing awareness and, potentially, consumption. The impact of marketing on adults, who account for most marketing exposure and consumption, should therefore both inform any debate about implementing such controls and be a cornerstone of evaluation.

The importance of examining population-level effects is becoming more prominent in alcohol marketing control literature. For example, evaluations of product warning labels in a Canadian province (Zhao *et al.*, 2020) and minimum unit pricing in Scotland (O’Donnell *et al.*, 2019) centered on adult consumers. The recent implementation of a suite of alcohol marketing controls in the Republic of Ireland (O’Dwyer, 2019) provides an ideal opportunity to generate real-world population-level data for a combination of marketing controls, including advertising placement restrictions, limits on the content of advertising communications and consumer protection and health messages on packaging, which would help to address key gaps in the literature (Siegfried *et al.*, 2014).

The related field of tobacco control also shows the wider benefits of population-level marketing control policies. This century, the UK Government has introduced a prohibition on tobacco advertising, promotion and sponsorship, a ban on the open display of tobacco products at the point-of-sale, standardized packaging for cigarettes and rolling tobacco and large pictorial health warnings on tobacco packaging (Action on Smoking and Health, 2020). All of these policies are targeted at, and intend to have an impact on, population-level smoking rates. The prevalence of adults in Great Britain (≥16 years) who are current smokers has fallen from 27.0% in 2000 to 15.8% in 2019 (Office of National Statistics, 2020), and evaluation evidence has demonstrated the positive impact of these specific policies on both adults (e.g. Harris *et al.*, 2006; Aleyan *et al.*, 2020) and young people (e.g. Moodie *et al.*, 2008; MacGregor *et al.*, 2020). This is not to suggest that employing identical legislative policies is necessarily the correct response for alcohol, but it is evident that it is appropriate, and indeed necessary, to view alcohol marketing through a population lens.

## Funding

N.C. was supported by a fellowship from the Society for the Study of Addiction (Stirling ID: 100879). C.M. received no funding for this research.

## Conflict of interest statement

N.C. is on the board of directors at Alcohol Focus Scotland and receives a Griffith Edwards Academic Fellowship from the Society for the Study of Addiction (SSA). C.M. has no conflict of interest to declare.
